# Clinical and haematological features of hyperhaemolysis in sickle cell disease: A case series from two tertiary care centres

**DOI:** 10.1111/bjh.70191

**Published:** 2025-10-06

**Authors:** Jaehyup Kim, James D. Burner, Christopher Webb, Ravi Sarode, Brian D. Adkins, Margaret Kypreos, Ibrahim F. Ibrahim, Yu‐Min Shen, Sean G. Yates

**Affiliations:** ^1^ Division of Transfusion Medicine and Hemostasis, Department of Pathology University of Texas Southwestern Medical Center Dallas Texas USA; ^2^ Division of Pulmonary and Critical Care Medicine, Department of Internal Medicine University of Texas Southwestern Medical Center Dallas Texas USA; ^3^ Division of Hematology, Department of Internal Medicine University of Texas Southwestern Medical Center Dallas Texas USA

**Keywords:** hyperhaemolysis, sickle cell disease

To the Editor,

Patients with sickle cell disease (SCD; including sickle cell anemia [HbSS] and related genotypes such as HbSC and HbSβ‐thalassemia) frequently require chronic RBC transfusions and are at high risk of delayed haemolytic transfusion reactions (DHTR). Although partial antigen matching has become standard practice to reduce alloimmunization rates in SCD patients, DHTR remains common due to several factors, including genotypic differences in RBCs between donor blood and recipients.^1^, ^2^, ^3^, ^4^ DHTR risk in SCD patients is reported to range from 1% to 19%,^5^ significantly higher than that of the general population, which is estimated to range from 1 in 1000 to 1 in 10 000.^6^, ^7^

A subset of SCD patients develops an exaggerated DHTR response known as hyperhaemolysis syndrome (HHS). This condition is thought to be caused by immune‐mediated haemolysis of both bystander autologous red blood cells (RBCs)^8^ and allogeneic transfused RBCs.^9^ Here, we describe the clinical and haematological characteristics of 10 SCD patients diagnosed with HHS at our institution. Additionally, we assess how interventions, including targeted monoclonal antibody therapies, affect outcomes. To our knowledge, this is the largest single‐centre case series of HHS in SCD reported to date.

This retrospective cohort study was conducted at the University of Texas Southwestern Medical Center Clements University Hospital and Parkland Memorial Hospital, both tertiary care hospitals, in Dallas, Texas, from 2020 to 2025. The study was approved by the University of Texas Southwestern Medical Center institutional review board (STU‐2020‐0068). Hyperhaemolysis was defined as a decline in haemoglobin (Hb) values below pre‐transfusion levels following the transfusion of red blood cells, accompanied by laboratory markers consistent with haemolysis (e.g. lactate dehydrogenase [LDH], bilirubin). Demographic data (age, gender, type of haemoglobinopathy and blood type), laboratory findings (Hb, % hemoglobin A (HbA), direct antiglobulin test [DAT], LDH, total bilirubin, haemoglobinuria and reticulocyte count) and clinical information (admission diagnosis, infection, treatment regimen) were collected through a retrospective chart review. The ratio of Hb was evaluated by two‐tailed Student's *t*‐test using GraphPad Software (GraphPad, Boston, MA, USA). No imputation was performed for missing data, and statistical significance was defined as a *p*‐value less than 0.05.

We identified a total of 10 patients diagnosed with HHS during the study period and described clinical features in Table 1, and laboratory characteristics in Table S1 for each patient. Table S2 summarizes the clinical characteristics of the study cohort. Immunohaematology findings varied, with two patients having a positive DAT. Patient 2 displayed pre‐ and post‐transfusion DAT positivity (polyspecific and Immunoglobulin G (IgG)) with a negative eluate, while patient 7 showed pre‐ and post‐transfusion DAT positivity (polyspecific and IgG) and a positive eluate for warm autoantibodies. Three patients (1, 6 and 7) had positive antibody screens both historically and currently, without developing new antibodies after hyperhaemolysis. Patient 8 had anti‐E, anti‐Fy^a^ and anti‐Jk^b^ before the transfusion of 10 units of RBCs during delivery and subsequently developed additional antibodies including anti‐C, anti‐K, anti‐S and warm autoantibodies. The median length of stay from hyperhaemolysis diagnosis was 9 days (Interquartile range (IQR) 6–11). Two patients (patients 4 and 6) died despite treatment, and no significant differences were observed in age, gender, time from transfusion to hyperhaemolysis, presence of antibodies, RBC transfusion type or length of hospital stay. Reports have suggested an association between severe acute respiratory syndrome coronavirus 2 (SARS‐CoV‐2) infection or vaccination and worsening haemolytic complications.^10^, ^11^ Three patients had confirmed SARS‐CoV‐2 infection or immunization within 1 month of hyperhaemolysis. Patient 1 received a vaccination 3 weeks prior to a red blood cell transfusion, while patients 4 and 9 tested positive for SARS‐CoV‐2 within 1 week of the transfusion. No association was observed between infection or vaccination and survival.

**TABLE 1:** Demographic information and outcomes of study subjects.

Patient number	Age (years)	Gender	Prior HHS	SARS‐CoV‐2 vaccine ≤1 month	SARS‐CoV‐2 positivity ≤1 month	Disease‐modifying agents	Indications for Tx	Type of Tx	Tx to HHS (days)	HHS treatment	LOS (days)	Outcome
1	40	F	Yes (2021)	Yes	N/A	Crizanlizumab, hydroxycarbamide	Preoperative optimization	RBCX	6	D/E/T/R	10	Survival
2	24	M	No	No	No	Hydroxycarbamide	ACS	Simple	9	D/E/T	11	Survival
3	21	F	Yes (2021)	No	No		Symptomatic anaemia	Simple	2	D/E	9	Survival
4	28	M	No	No	Yes	Hydroxycarbamide	Priming for TPE for multi‐organ failure	Simple	0	D/E	3	Death
5	36	M	Yes (2016)	No	No		Symptomatic anaemia	Simple	0		3	Survival
6	50	M	No	No	No	Voxelotor, crizanlizumab	ACS	Simple	9	D/E/T	7	Death
7	21	M	Yes (2020)	N/A	No		Sickle hepatopathy	RBCX	6	E	31	Survival
8	36	F	No	No	No		Postpartum haemorrhage	Simple	7	D	9	Survival
9	30	F	Yes (2024)	No	Yes	Hydroxycarbamide	Pain crisis	Simple	6	T	6	Survival
10	19	M	No	N/A	No		ACS	RBCX	2	D/E/T	43	Survival (stroke)

Abbreviations: ACS, acute chest syndrome; D, darbepoetin; E, eculizumab; HHS, hyperhaemolysis syndrome; LOS, length of stay; R, rituximab; T, tocilizumab; TPE, therapeutic plasma exchange.

The Hb levels and the ratio of Hb calculated by dividing the Hb value by that of the previous day for the first 10 days are summarized in Table 2. The Hb level at the time of hyperhaemolysis diagnosis did not appear to differ significantly between survivors and non‐survivors (median 5.8 vs. 5.3 g/dL). However, the Hb ratio for the first 3 days showed a statistically significant difference between the survival and non‐survival groups (0.94 ± 0.16 vs. 0.73 ± 0.15, *p* = 0.03). A Hb ratio of ≤0.8 or an absolute daily Hb drop of ≥1.5 g/dL identified non‐survivors with a sensitivity of 100% and a specificity of 75% for this case series, with a Youden index of 0.75. Four patients (patients 1, 4, 6 and 10) experienced a rapid Hb drop, defined as a Hb ratio of 0.8 or an absolute daily Hb decline of ≥1.5 g/dL (bold italic in Table 2). Darbepoetin and eculizumab were started on the day of rapid Hb drop for patient 1, who subsequently recovered. Eculizumab therapy started on the day of rapid Hb drop for patient 4, but the patient continued to experience Hb decline and expired. Patient 6 received only steroids and intravenous immunoglobulin (IVIG) on the day of rapid Hb drop and expired. Patient 10 started receiving darbepoetin on the day of rapid Hb drop and recovered from hyperhaemolysis, although the clinical course was complicated by an embolic cerebral stroke caused by fat emboli. Hb and LDH trends for individual patients, along with the treatments used, are summarized in Figure S1.

**TABLE 2:** Haemoglobin (Hb) level and ratio for subjects over the first 10 days.

Patient number	1		2		3		4^a^		5		6^a^		7		8^c^		9^c^		10	
	Hb	Hb ratio	Hb	Hb ratio	Hb	Hb ratio	Hb	Hb ratio	Hb	Hb ratio	Hb	Hb ratio	Hb	Hb ratio	Hb	Hb ratio	Hb	Hb ratio	Hb	Hb ratio
Admission	8.8		3.4		8.2		11.1		7.8		7.0		7.2		6.0		8.3		11.3	
Day −1	8.8		5.3		6.2^b^		6.3		4.2		6.9		7.7		6.0		8.6		7.0	
Day 0	7.3		3.4		4.3		3.9		4.2		6.7		6.4		5.2		7.5		7.2	
Day 1	***4.1***	***0.56***	3.3	0.97	3.7	0.86	***2.1***	***0.54***	3.8	0.90	6.2	0.93	5.8	0.91	4.8	0.92	6.7	0.89	***4.7***	***0.65***
Day 2	4.2	1.02	3.2	0.97	3.4	0.92	1.7	0.81	3.6	0.95	***4.7***	***0.76***	4.9	0.84	4.5	0.94	5.4	0.81	4.0	0.85
Day 3	4.2	1.00	3.2	1.00	3.6	1.06			5.2	1.44	***3.0***	***0.64***	4.7	0.96	4.6	1.02	5.9	1.09	4.0	1.00
Day 4	3.9	0.93	2.9	0.91	3.3	0.92					2.8	0.93	5.3	1.13	4.3	0.93	5.3	0.90	3.8	0.95
Day 5	3.9	1.00	3.3	1.14							2.8	1.00	5.3	1.00	4.9	1.14	5.4	1.02	3.9	1.03
Day 6	4.6	1.18	3.4	1.03							2.6	0.93	5.1	0.96	5.2	1.06			3.8	0.97
Day 7	5.1	1.11	4.2	1.24	5.7						2.2	0.85	5.4	1.06	6.1	1.17			4.2	1.11
Day 8	5.2	1.02	4.4	1.05									5.6	1.04	6.6	1.08			4.2	1.00
Day 9	5.7	1.10			6.4								6.0	1.07	6.6	1.00			4.1	0.98

*Note*: Bold Italics: Hb ratio <0.8 or Hb level decrease >1.5 g/dL over 1 day.

Abbreviations: Hb, haemoglobin level in g/dL; Hb ratio, current Hb divided by Hb of previous day.

^a^ Non‐survival.

^b^ Day −2 Hb.

^c^ Transfusion performed at an outside hospital prior to admission.

The efficacy of monoclonal antibodies targeting immune pathways in severe or recalcitrant cases of hyperhaemolysis has been reported by several studies and may serve as useful adjunct therapies.^12^, ^13^ However, criteria for when to consider these second‐line therapies do not exist. This study compared laboratory and clinical data between survivors and non‐survivors and found a rapid early decline in Hb as a potential marker of poor prognosis, which could help identify high‐risk patients who may benefit from early administration of second‐line therapy for hyperhaemolysis, including erythropoietin mimetics and monoclonal antibody therapy. Pretreatment with disease‐modifying agents, such as hydroxycarbamide (hydroxyurea) and crizanlizumab, or the use of HHS treatments, such as rituximab, eculizumab or tocilizumab, did not have a clear association with survival. Three of the patients had SARS‐CoV‐2 infection or vaccination within 1 month of transfusion, with the hospital course of patient 4 previously described in a published case report.^11^ The potential role of SARS‐CoV‐2 in accelerated RBC turnover^14^ or increased secretion of pro‐inflammatory cytokines following SARS‐CoV‐2 vaccination^15^ raises the possibility that SARS‐CoV‐2 may serve as a trigger for hyperhaemolytic episodes. However, it is unclear whether the vaccination or infection rate of SARS‐CoV‐2 differs between patients with hyperhaemolysis and the general population. Further research into the role of SARS‐CoV‐2 as a trigger for hyperhaemolytic episodes may be worthwhile.

While this case series is one of the larger ones focused on hyperhaemolysis, comprised of 10 patients, the number of patients may not be sufficient to define absolute Hb drop or Hb ratio for high‐risk patients. We also noted a significant variation in clinical management between patients, which complicated the analysis of the effect of treatment regimens. Further studies involving a larger patient population across multiple institutions could refine the proposed threshold more accurately, enabling targeted early use of second‐line therapies.

## FUNDING INFORMATION

No funding to report.

## AUTHOR CONTRIBUTIONS

Jaehyup Kim was responsible for the conceptualization of research, research design, data collection, data analysis and manuscript writing. James D. Burner was responsible for manuscript writing. Christopher Webb was responsible for data collection and manuscript writing. Ravi Sarode was responsible for manuscript writing. Brian D. Adkins was responsible for manuscript writing. Margaret Kypreos was responsible for manuscript writing. Ibrahim F. Ibrahim was responsible for manuscript writing. Yu‐Min Shen was responsible for manuscript writing. Sean G. Yates was responsible for the conceptualization of research, research design, data collection, data analysis and manuscript writing.

## CONFLICT OF INTEREST STATEMENT

The authors have no conflicts of interest to disclose.

## Supporting information


**Table S1.** Laboratory results, indication and nature of RBC transfusion, treatment and hospital course. *: Transfusion occurred at an outside hospital. The pretransfusion level is from an outpatient clinic visit.
**Table S2.** Summary of patient characteristics.

**Figure S1.** The trend of haemoglobin and LDH and the administration schedule of the treatment regimen.

## Data Availability

Data are available upon request.
