# Supplementary material for: Clinical and haematological features of hyperhaemolysis in sickle cell disease: A case series from two tertiary care centres
Source: Br J Haematol. 2025 Oct 6;207(6):2643–7. doi: 10.1111/bjh.70191 (PMC12710111; doi:10.1111/bjh.70191)
Supplement: Supplementary file 1 — Table S1. Laboratory results, indication and nature of RBC transfusion, treatment and hospital course. *: Transfusion occurred at an outside hospital. The pretransfusion level is from an outpatient clinic visit. Table S2. Summary of patient characteristics. [file BJH-207-2643-s001.docx]

| **Patient number** | **Blood Type** | **Genotype** | **Preexisting alloantibodies** | **Pre-Tx platelet (103/ul)** | **Pre-Tx WBC (103/ul)** | **Pre-Tx Hb (g/dL)** | **Post-Tx Hb (g/dL)** | **HHS DX Hb (g/dL)** | **DAT** | **Outcome** |
| --- | --- | --- | --- | --- | --- | --- | --- | --- | --- | --- |
| 1 | B+ | HbS/Bo | anti-C, E, Jkb, V | 124 | 2.76 | 7.8 | 10.6 | 4.1 | Negative | Survival |
| 2 | O+ | HbSS | negative | 173 | 17.8 | 3.4 | 5.3 | 3.4 | Positive (Pre and Post, negative eluate) | Survival |
| 3 | B+ | HbSS | negative | 337 | 27.63 | 4.7 | 6.2 | 4.3 | Negative | Survival |
| 4 | B+ | HbSS | negative | 68 | 9.62 | 5.8 | 6.3 | 3.9 | N/A | Death |
| 5 | B+ | HbSS | negative | 80 | 16.9 | 3.9 | 4.2 | 3.8 | Negative | Survival |
| 6 | O- | HbSS | anti-E, Jsa, M | 398 | 13.08 | 5.9 | 6.9 | 4.7 | Negative | Death |
| 7 | O+ | HbSS | HTLA, anti-Jkb, WAA | 250 | 20.49 | 7.1 | 11 | 6.4 | Positive (Pre and Post, WAA) | Survival |
| 8 | O+ | HbSC | anti-C, E, Fya, Jkb, K, S, WAA | 273 | 8.36 | 9.9 | N/A* | 5.2 | Negative | Survival |
| 9 | O+ | HbSS | negative | 195 | 7.7 | 7.2 | 7.9 | 6.7 | N/A | Survival |
| 10 | B+ | HbSS | negative | 44 | 17.27 | 6.9 | 10.9 | 4.7 | N/A | Survival (Stroke) |

**Supplementary Table 1.** Laboratory results, indication, and nature of RBC transfusion, treatment, and hospital course. *: Transfusion occurred at an outside hospital. The pretransfusion level is from an outpatient clinic visit. (Tx: Transfusion, WBC: White blood cell, DAT: Direct agglutination test, HTLA: High titer low avidity antibody, WAA: Warm autoantibody )

| **Characteristics** |  | **Value** |
| --- | --- | --- |
| Age (Years) | Range (Median) | 19-50 (29) |
| Gender | Female | 4 |
|  | Male | 6 |
| Hemoglobinopathy | HbSS | 8 |
|  | HbSC | 1 |
|  | HbS/beta-thalassemia | 1 |
| Presence of alloantibodies before transfusion | | 4 |
| Prior episode of Hyperhaemolysis | | 5 |
| SARS-CoV-2 exposure in 1 month | Vaccination | 1 |
|  | Active infection | 2 |
| Disease-modifying agent | Voxelotor | 1 |
|  | Crizanlizumab | 2 |
|  | Hydroxyurea | 4 |
| Type of Transfusion | Simple | 7 |
|  | RBC exchange | 3 |
| Transfusion to Hyperhaemolysis (Days) | Range (Median) | 0-9 (6) |
| Hyperhaemolysis treatment | Steroids | 10 |
|  | IVIG | 10 |
|  | Darbepoetin | 7 |
|  | Eculizumab | 7 |
|  | Tocilizumab | 5 |
|  | Rituximab | 1 |
| Outcome | Survival | 8 |
|  | Death | 2 |

**Supplementary Table 2.** Summary of patient characteristics


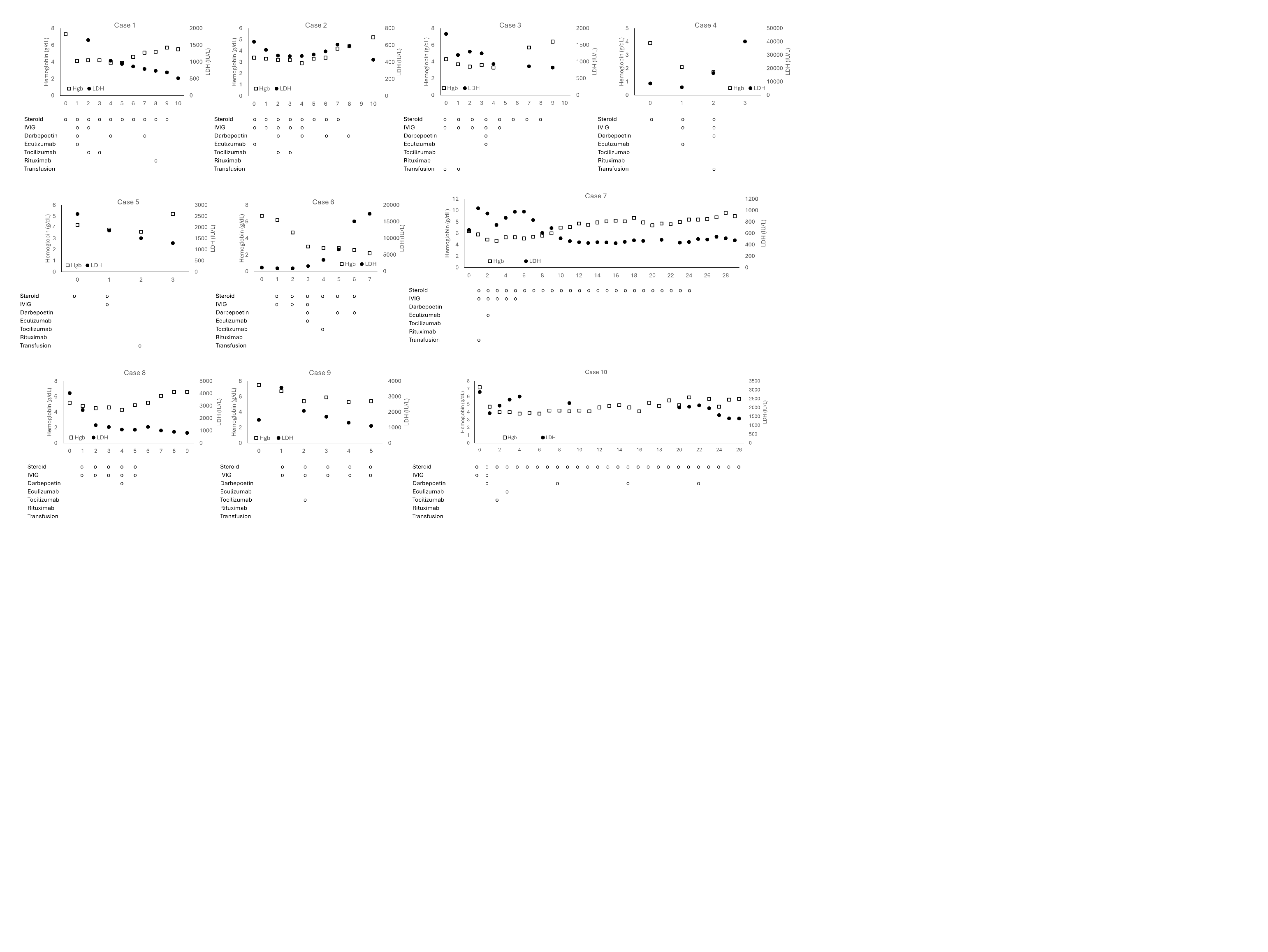


**Supplementary Figure 1**. The trend of hemoglobin and LDH and the administration schedule of the treatment regimen.
